# Low-Power Transit Time-Based Gas Flow Sensor with Accuracy Optimization

**DOI:** 10.3390/s22249912

**Published:** 2022-12-16

**Authors:** José R. García Oya, Alejandro Sainz Rojas, Daniel Narbona Miguel, Ramón González Carvajal, Fernando Muñoz Chavero

**Affiliations:** Electronic Engineering Department, University of Seville, E-41092 Seville, Spain

**Keywords:** cross-correlation, IoT sensors networks, piezoelectric transducers, transit time measurement, ultrasonic gas flow sensors

## Abstract

In this paper, a fully designed ultrasonic transit time-based gas flow sensor is presented. The proposed sensor has been optimized in terms of accuracy, sensitivity, and power consumption at different design stages: mechanical design of the sensor pipe, piezoelectric transducer configuration and validation over temperature, time of flight detection algorithm, and electronics design. From the optimization and integration of each design part, the final designed gas flow sensor is based on the employment of 200 kHz-piezoelectric transducers mounted in a V-configuration and on the implementation of a cross-correlation algorithm based on the Hilbert Transform for time-of-flight detection purposes. The proposed sensor has been experimentally validated at different flow rates and temperatures, and it fully complies with the accuracy specifications required by the European standard EN14236, placing the proposed design into the state of the art of ultrasonic gas flow sensors regarding cost, accuracy, and power consumption, the latter of which is crucial for implementing smart gas meters that are able to autonomously operate as IoT devices by extending their battery life.

## 1. Introduction

Typically, mechanical sensors have been the usual choice for implementing gas meters. However, due to the current low cost, high-energy efficiency, and interoperability requirements in facilities, more suitable technologies to be integrated into smart meters have been explored, such as those based on ultrasonic transmission, which has been widely studied in recent years [1,2,3].

Ultrasonic gas flow sensors allow for longer maintenance periods, because they are less affected by wear, and they can maintain higher accuracy throughout their useful life. Moreover, the flow meter does not have direct contact with the gas, thus preventing corrosion or deterioration of the sensor. Additionally, they are non-invasive sensors (avoiding a pressure drop due to their own installation or obstruction in the flow), the composition of the gas inside of the meter could be unknown (because the measurement is independent of the theoretical speed of sound), and additional information of gas properties (such as sound velocity profile) can be used for density and calorific value determination. Moreover, the use of a filter is not required to protect the sensor (leading to a lower cost in the installation), and they have capabilities of self-diagnosis to validate their correct operation [4].

In addition, ultrasonic sensors are implemented with almost purely electronic elements, so this technology can exploit the benefits from the advances in ultra-low consumption that are available today, with the aim of extending the battery life in the same order of time (more than 10 years), and then the accuracy specifications of the ultrasonic sensor can be maintained. Likewise, ultrasonic sensors based on electronic designs facilitate the management of different operation modes in order to optimize their energy consumption and operating times. Therefore, smart meters based on this technology will allow connectivity from remote stations to the central server, facilitating correct billing and the implementation of a sensor network under the Internet of Things (IoT) paradigm for domestic and remote sensing applications [5,6,7,8,9,10,11]. This will not only help to reduce the time spent collecting data, but it will also improve resource allocation, eliminating erroneous readings and the need for manual monitoring, generating added value for utility companies and facilitating a broader spectrum of data to better serve customers. This automatic data collection includes those data related to consumption and also those related to the self-diagnosis of the sensor, which are transferred to the central system for billing, troubleshooting, and analysis. Of course, ultrasonic gas flow sensors have some limitations, which can affect the accuracy measurement, such as a susceptibility to the interferences of bubbles, uncertainty in the installation process, or dependency of the piezoelectric transducers on temperature variations.

Although several ICs (integrated circuits) have recently emerged in the market, which provide integrated ultrasonic front ends and cost-effective solutions, it is necessary to determine several design parameters at the mechanical, electronic, and signal-processing level, in order to optimize the ultrasonic flow sensors. Therefore, this paper presents a complete set of ultrasonic flow sensor guidelines in order to experimentally select each appropriate component, such as the employed IC, the detection algorithm, the piezoelectric transducer frequency, the transducer placement on the pipe, and the sensor’s mechanical dimensions, with each decision oriented towards enhancing accuracy and power consumption.

This work is mainly motivated by the different technical challenges to be addressed in order to fully design an ultrasonic gas flow sensor, as the previously published works have usually focused on accuracy optimization at only one design level. In this way, in [2], accuracy is optimized from a novel hybrid mechanical configuration, increasing the cost per unit with the employment of four piezoelectric transducers, and implemented using a high-cost high-energy device, such as a Field Programmable Gate Array (FPGA). In [3], accuracy is enhanced by the implementation of a novel least-squares (LS)-based time-of-flight (ToF) detection algorithm focused on low SNR environments, but at the expense of increasing its computational burden, requiring it to be implemented by an external computer and so preventing its use in domestic applications. On the other hand, there are previously published works based on low-cost electronics, such as [1], which achieves adequate accuracy as well, although it only provides measurements at an ambient temperature, without the ability to compensate for thermal drift, as well as without providing power consumption results. These publications are compared with the present work in terms of accuracy in Section 3.

Thus, the sensor presented in this paper is focused on the optimization of accuracy by the appropriate analysis, selection, and integration of each design part of the sensor. It still reduces the hardware cost and power consumption, which are usually not described in the previously published works. As such, we provide an integrated solution that encompasses all of these design requirements to be employed in domestic applications.

The emergence of new commercial off-the-shelf (COTS) ultrasonic front ends requires a thorough design procedure that simultaneously considers accuracy, power consumption, and cost. As mentioned, so far, the previous literature has been focused on improving specific aspects of the design. However, the purpose of this paper is to address the design of an ultrasonic gas flow sensor from scratch by successively considering all aspects of the design:

1. Detection algorithm and selection of its parameters based on accuracy and power consumption criteria.
2. Selection and design of required hardware.
3. Mechanical design optimization in terms of accuracy.
4. Transducer selection and characterization.

In summary, this paper provides that which is lacking in previously published works, i.e., detailed information on all aspects necessary to reproduce the design of a fully operational ultrasonic gas flow sensor, starting from a commercial front-end.

As result of the present research, this paper proposes a high-accuracy ultrasonic gas flow sensor for energy-efficient applications in gas distribution networks, reducing the power consumption and cost per unit, which is crucial for the idea of massive distribution networks. The proposed gas flow sensor is based on a V-configuration pipe (with an ultrasonic length of 61.1 mm, section of 194.75 mm^2^, and incidence angle of 65°), using 200 kHz-piezoelectric transducers and a cross-correlation method based on the Hilbert Transform.

The paper is organized as follows. Section 2 describes the fundamental principles of the selected technology and the different stages of the complete design of the sensor. In Section 3, with the integration of the previously described design parts, the final experimental accuracy and power consumption results are discussed and compared with several related works. Finally, the main conclusions are detailed in Section 4.

## 2. Materials and Methods

### 2.1. Fundamentals Principles

The measurement principle of ultrasonic flow sensors is based on the transmission and reception of an acoustic wave through the medium, employing two piezoelectric transducers installed on the pipe. They can be classified into transit-time or Doppler effect sensors [4].

**Figure 1:**
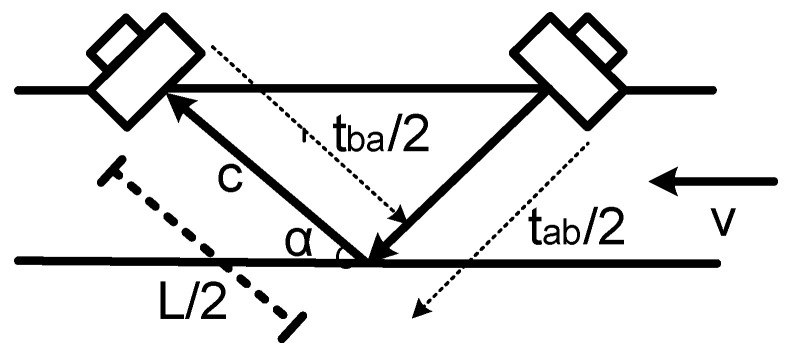
Measurement principle.

Transit-time sensors measure the difference in propagation times of ultrasonic pulses upstream and downstream, as illustrated in Figure 1. Absolute ToF are given by Equations (1) and (2), where *L* is the distance of the ultrasonic path, *c* is velocity of the sound in the medium, *v* is the flow velocity, and *α* is the incidence angle [12]. From Equation (3), it can be observed how it is possible to measure v without dependency with the medium, so it will be possible to measure the flow rate *Q* (by using Equation (4)) from the average of v and the pipe cross section *S*. In addition, from Equation (5), it is possible to measure *c* without dependency with the flow velocity [12]. This property can be employed as a basis to compensate for the flow measurement errors due to temperature variations, from an accurate measurement of *t_ab_* and *t_ba_*, i.e., *c*. The dependency of *c* with the temperature *T* is given by Equation (6), where *γ* is the adiabatic index, *M* is the molar mass of gas, and *R* is the universal gas constant [13].

(1) $$t_{ab}=\frac{L}{c+v\cdot \cos(\alpha )},$$

(2) $$t_{ba}=\frac{L}{c-v\cdot \cos(\alpha )},$$

(3) $$v=\frac{L}{2\cdot \cos(\alpha )}\cdot \left(\frac{1}{t_{ab}}-\frac{1}{t_{ba}}\right),$$

(4) $$Q=\bar{v}\cdot S,$$

(5) $$c=\frac{L}{2}\cdot \left(\frac{1}{t_{ab}}+\frac{1}{t_{ba}}\right),$$

(6) $$c=f(T)=\sqrt{\frac{\gamma \cdot R\cdot T}{M}}$$

Additionally, from Equation (3), the theoretical differential ToF (DToF) can be extracted as:

(7) $$\Delta =\frac{Q\cdot t_{ab}\cdot t_{ba}\cdot 2\cdot \cos(\alpha )}{S\cdot L_{}}.$$

From Equation (7), it is possible to define the flow rate as:

(8) Q=kg⋅Δtab⋅tba,

where *k_g_* = *S*·*L*/2·cos(*α*).

Alternatively, another common flow measurement method using ultrasonic technology is based on the measurement of the phase shift between the transmitted and the received signals. However, this method can only be used for distances up to the ultrasonic wavelength, in order to avoid measurements ambiguities [14]. For example, for gas applications, working in a common frequency of 200 kHz, this distance between transducers should be around 2–3 mm, which is insufficient, as described in Section 2.2.2. Thus, for distances longer than the wavelength, the usual method is based on the measurement of DToF.

On the other hand, Doppler effect sensors are based on the measurement of the difference between the frequencies of both transmitted acoustic signals. The interpretation of this measurement is similar to that of transit-time sensors, with the variation in the wavelength proportional to the flow velocity in this case. In order for Doppler-based flow sensors to work properly, there must be a concentration of solid particles or air bubbles flowing in the medium, because the movement of the particles changes the frequency of the ultrasonic signal, so the flow sensor can measure this frequency shift, which is linearly proportional to the flow rate. Therefore, Doppler-based sensors are more suitable for measuring flows of dirty or aerated liquids, such as sewage and sludge, whereas transit time-based sensors (selected in this work) are more appropriate for gases and clean liquids, such as drinkable water or oil.

### 2.2. Ultrasonic Flow Sensor Design

The design methodology of the selected transit time-based sensor has been deduced from the five design stages described in this section, in order to optimize ToF measurement accuracy and power consumption. These design stages are the following:

1. Selection of the ToF detection algorithm and the low-cost hardware in which it will be implemented.
2. Design of the different sensor pipes, with different materials, dimensions, and transducer configurations, which are compared in terms of accuracy.
3. Selection and validation of piezoelectric transducers at different nominal frequencies, which are experimentally tested at different temperatures and flow conditions.
4. Sensor configuration, i.e., selection of the different parameters for transmission and reception of the ultrasonic signal.
5. Design of the electronic circuitry to properly generate, receive, filter, and amplify the ultrasonic signals.

**Figure 2:**
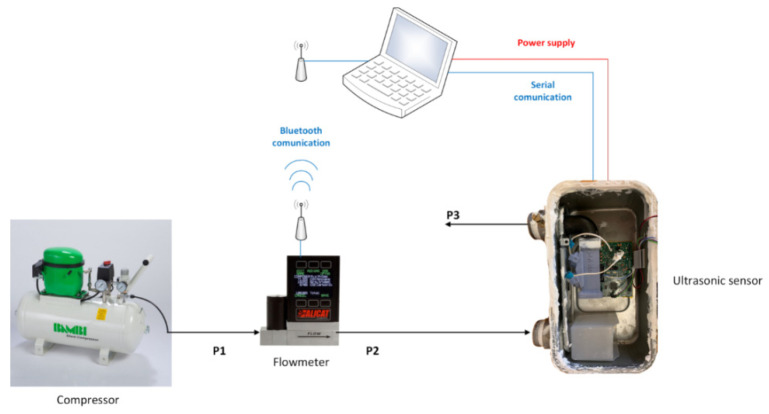
Experimental setup.

Note that uncertainty results revealed in this section are given by:

(9) $$U=\frac{\sigma_{Q}}{Q_{av}}\cdot 100\%,$$

where *σ_Q_* is the standard deviation, and *Q_av_* is the average flow rate by performing one measurement each 500 ms for a set measurement duration of 3 min. For this purpose, the tests described in this paper are performed using an experimental setup, based on a compressor and three different high-accuracy flow meters as benchmark measurements for three different flow ranges (models MC-10SLPM-D-DB9M/CM, MCP-100SLPM-D-DB9M/CM, MCR-250SLPM-D/CM from the Alicat manufacturer), as illustrated in Figure 2. This experimental setup was implemented at room temperature (23 °C) and also at all the temperature ranges (−10° to 40 °C) by using a climatic chamber, for the specific tests described in Section 2.2.3. Additionally, in Figure 2, pressure at different points of the setup is illustrated as well, where *P1* = 3 bars, *P3* = 1 bar, and *P2* depends on the flow, resulting at around 1.3 bars at 2000 L/h. Finally, Figure 2 also includes the serial communication to collect the measurement data and the Bluetooth communication, with the flow meter used as reference.

#### 2.2.1. ToF Detection Algorithm and Hardware Selection

The main methods to implement transit time-based gas flow sensors are based on: (1) time-to-digital (TDC), based on the detection of the zero crossings of the received signal; and (2) analog-to-digital (ADC), based on the capture of the whole received waveform. In other words, for delay estimation purposes, TDC solutions are based on the use of local properties of the received signals, whereas ADC solutions are based on the use of global properties. The ADC-based solution provides a better accuracy than TDC-based methods, because it is possible to implement the cross-correlation between both received signals, performing a noise digital filter and enhancing the zero-flow drift performance. Finally, the ADC-based solution is more robust regarding signal amplitude variations in cases of high flow rates, transducer-to-transducer deviation, and temperature variations, providing additional information regarding the degradation of the sensor, which is crucial for this long-life sensor application. Of course, this ADC-based option, selected for this application, implies a higher power consumption in terms of signal processing time. Otherwise, a TDC-based option implies a higher power consumption in terms of excitation voltage, because this solution needs a higher amplitude at the pulse generator output. In any case, the enhanced accuracy achieved with the proposed ADC-based solution will allow for a lower number of ToF measurements, each at 2 s, which is the flow measurement time specified by the standard EN14236.

Note that the ADC-based approach could be also implemented as a first approximation by a conventional threshold method, which is based on the detection of a received amplitude higher than this threshold level in order to estimate ToF. However, this simpler method usually presents errors that cannot be compensated for when the amplitude of the received signal is not constant with distance [15]. Thus, a ToF estimation based on correlation leads to a more suitable option, consisting of the time detection at this correlation reaching its maximum, and presenting a better accuracy performance than the threshold technique, especially for low signal-to-noise (SNR) scenarios [16,17,18,19,20].

Moreover, for this implementation, the implementation of the Hilbert Transform (HT) over the original received signal has been selected as the method to obtain the envelope of the received signals and also to improve the accuracy for ToF estimation. The HT of a real signal is given by Equation (10) [21]:

(10) $$\tilde{x}(t)=H[x(t)]=\frac{1}{\pi }{\int^{\;}}_{-\infty }^{\infty }\frac{x(u)}{\left(t-u\right)}du.$$

Thus, the HT of a real signal *x*(*t*) is the convolution *x*(*t*) * (1/*πt*). This transform returns an imaginary part which, combined with the real part, can be used to obtain an analytic signal such as:

(11) $$x_{a}(t)=x(t)+jH[x(t)]=x(t)+j\tilde{x}(t).$$

Finally, the envelope of the signal *x*(*t*) can be obtained from the modulus of this analytic signal:

(12) $$\left|x_{a}(t)\right|=\sqrt{x^{2}(t)+{\tilde{x}}^{2}(t)}.$$

Once both envelopes have been obtained (for the received signals *x*(*t*) and *y*(*t*)), cross-correlation is implemented with the objective of estimating the ToFs from the detection of its peak position [22,23]. Additionally, the HT of the signal *y*(*t*) can be employed for the implementation of a cross-correlation algorithm instead of using the signal *y*(*t*) itself (CCF). The cross-correlation function obtained with the Hilbert Transform (CCFHT) is equal to:

(13) $$R_{x\tilde{y}}(\tau )=CCF[x(t)\tilde{y}(t+\tau )].$$

**Figure 3:**
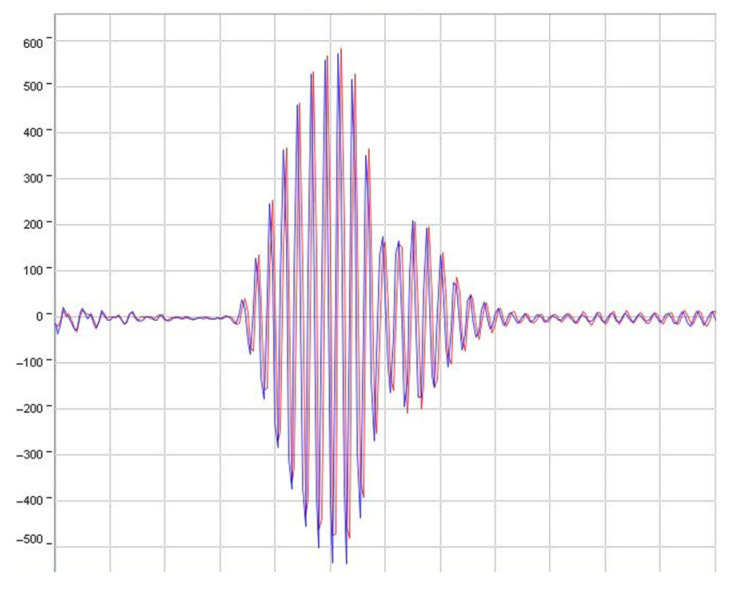
Received signal waveforms.

Then, a zero-crossing detection is used to correct the peak position in order to obtain a subsampled delay time *τ* (i.e., DToF for this application) estimation. This method also leads to a simpler implementation, by computing a linear interpolation [24] instead of the parabolic or Gaussian interpolations generally used for the CCF case [21], and so allowing for a lower energy implementation. In any case, since it is only possible to achieve an accuracy limited by the sampling rate *f_s_* [25] (since ToF = *n*/*f_s_*, where *n* is the sample where the maximum or zero is detected) when using a coarse ToF estimation from the cross-correlation, a subsample accuracy stage has to be performed, such as the linear interpolation over the CCFHT implemented for this approach. Finally, Figure 3 illustrates both received signals’ waveforms.

Finally, once an ADC correlation-based method using the HT for ToF estimation has been selected, the core of the electronic circuitry for its implementation will be based on COTS, in order to perform a compact, robust, integrated, and cost-effective solution. Specifically, it is based on the TI MSP430FR6043 component. This device has been selected because it provides a complete analog front-end for the ultrasonic link, based on a pulse generator in transmission and a Programmable Gain Amplifier (PGA) and an ADC in reception, by using an internal 16-bit RISC microprocessor with capabilities to implement the selected cross-correlation based algorithm. Additionally, the selected integrated circuit will reduce the cost per sensor unit and will also provide a communication interface with the customer board, i.e., the gas flow metering unit.

#### 2.2.2. Mechanical Design

At the mechanical level, the sensor has been optimized in terms of the phase difference between ultrasonic links, sensitivity, and flow stabilization.

As a first general design consideration, a pipe with a rectangular cross-section was selected, because with a rectangular shape, it can be demonstrated how the phase difference between ultrasonic paths is reduced [26], leading to a flow measurement under more similar conditions for upstream and downstream ultrasonic wave propagation paths.

Regarding the material employed for the pipes, different compositions were preliminary manufactured (using 3D printers) and tested, because the imperfections of the pipe, such as internal wall roughness or inhomogeneity in materials, can affect the flow measurement accuracy [27,28], which can also be affected by the capacity of the material to clamp the transducers. Thus, it will be necessary to avoid their misplacement, which could lead to possible uncertainties, as modeled in [29,30]. After this preliminary analysis, a photopolymer resin was selected, because its features of homogeneity, rigidity, and stability regarding climatic changes provided better accuracy results than other tested materials such as PLA (poly-lactic acid), SLA (stereo-lithographic), or ABS (acrylonitrile butadiene styrene).

**Figure 4:**
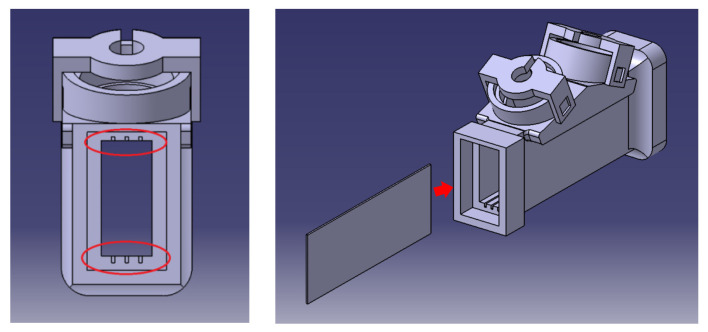
Installation of the metallic plates.

Finally, for flow stabilization purposes, metallic planar separation plates have been installed into the flow path pipe in order to make the flow velocity distribution profile more uniform. Specifically, three equally spaced metallic plates, with a thickness of 0.5 mm, have been installed in the pipe, as illustrated in Figure 4, where it is possible to appreciate the 6 rails where the plates are fixed.

**Figure 5:**
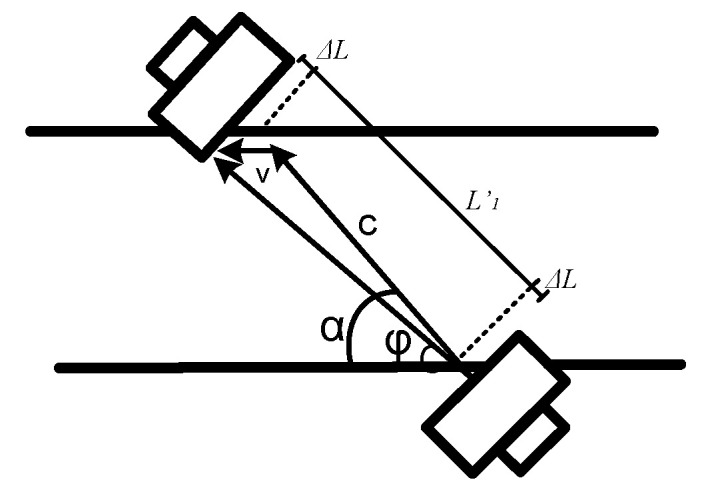
Ultrasonic beam deviation.

From these preliminary considerations, the configurations of two ultrasonic paths were tested: a Z-configuration (based on a direct route between the transducers) and a V-configuration (based on one reflection of the ultrasonic wave over the pipe floor). For each configuration, different form-factor alternatives have been analyzed, i.e., different incidence angles, cross-sections, and ultrasonic path lengths. For this mechanical analysis based on geometric variations, the starting point is based on the known values of the velocity of the sound *c* (435 m/s, calculated from the measurement of absolute ToF at zero-flow conditions), and the flow rate range is given by the standard (40–7200 L/h). The target parameters will be the flow velocity *v* and the DToF ranges. From Equation (4), modifying the cross-section *S,* it is possible to obtain different ranges for *v*. Its maximum should be limited, because higher values of *v* will provoke a higher deviation of the ultrasonic beam, as shown in Figure 5, causing different ADC signal levels in the upstream and downstream direction for high flow rates [31]. Note that although an along-beam configuration (based on a direct route between the transducers in parallel with the flow direction) is less affected by the beam deviation and non-reciprocity than the Z and V configurations [32], these effects have been considered by the appropriate selection of the values of *L*, *α,* and *S*, in the function of the transducer’s diameter and the beam’s deviation at the maximum flow rate (7200 L/h), as described below.

On the other hand, a higher value of the minimum DToF will improve accuracy at low flow rates, because SNR would be increased. However, lower values of *v* imply lower values of DToF, so this means a design trade-off: reducing *S* improves accuracy at low flow ranges, but it increases the sonic beam deviation caused by a higher *v*. Thus, knowing these target parameters, the main steps followed by the design are:

1. The ultrasonic path length is modified assuming this trade-off: a longer length leads to a higher interaction between the ultrasonic signal and the flow gas, increasing accuracy; however, the signal attenuation will be increased as well.
2. The incidence angle *α* is modified as well, implying different path lengths.
3. The cross-section *S* = *W·H* is modified, with the width *W* and the height *H*, where *H* = *L*1′*·sin*(*α*) (for a Z-scheme), *H* = *L*1′*·sin*(*α*)/2 (for a V-scheme), and *H* = *L*1′*·sin*(*α*)/4, with *L*1′ *= L*1–2*·*Δ*L*, where *L*1′ is the path length inside the pipe, and Δ*L* the distance between the pipe and the transducer (Figure 5).

**Figure 6:**
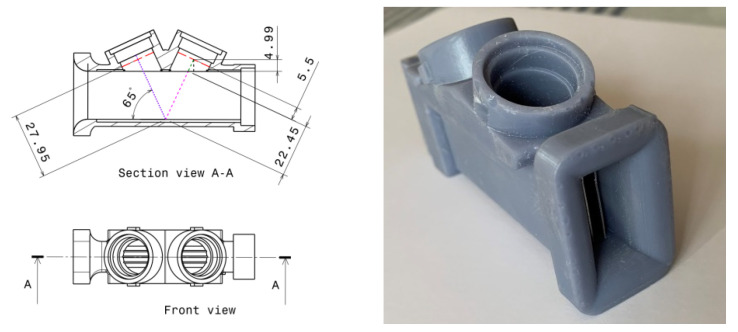
Designed and manufactured V2 pipe.

For each trio of values (*L*1*-α-S*), the *v* range is calculated from Equation (4), the absolute ToF ranges from Equations (1) and (2), and the DToF from Equation (7). In order to analyze different alternatives for the minimum DToF (at 40 L/h) and the maximum *v* (at 7200 L/h), a set of mechanical designs was performed. A list with the main manufactured and tested designs is shown in Table 1. As an example, Figure 6 illustrates the designed and manufactured V2 pipe.

The first step to size these designs consisted of performing a sweep of *α* in order to obtain its value, which provokes a minimum deviation of the ultrasonic beam for the worst case (at 7200 L/h), by using trigonometric calculations from Figure 5. Minimum Δ*x* deviation values were achieved with *α* ≈ 65°, resulting in Δ*x* ≈ 0.6mm, which seems small enough compared with the selected transducer diameter of 14.1 mm (described in Section 2.2.3). The mechanical designs listed in Table 1, for the case of a V-configuration, consist of a preliminary design (V1), with *α* = 45° (in order to test the effect of a higher Δ*x*) and several designs in the range of *α* = 63–67°. Note that most of designs were manufactured with *α* = 65°, because it presented a better accuracy of results than the cases of *α* = 63° (V3) and *α* = 67° (V4). Designs with *α* = 65° (V2, V5–V7) were manufactured with slightly combined variations of *L*, *H,* and *W* in order to test their influence on signal attenuation, interaction of the ultrasonic wave with the gas, and ultrasonic beam deviation effects.

Alternatively, several Z-designs were performed for different values of *L*1*-α-S* in the range of V-designs. The results with the best accuracy were achieved for Z1 and Z2, included in Table 1 as the most representative cases. However, the accuracy of these results is still lower than all the V-designs, so a Z-configuration was finally discarded. In addition, a V-configuration will allow for compensating for possible flow disturbances by the ultrasonic wave propagation along the reciprocate paths [2]. Finally, a V-configuration allows for a simpler assembly, because both transducers are placed at the same side of the pipe.

**Figure 7:**
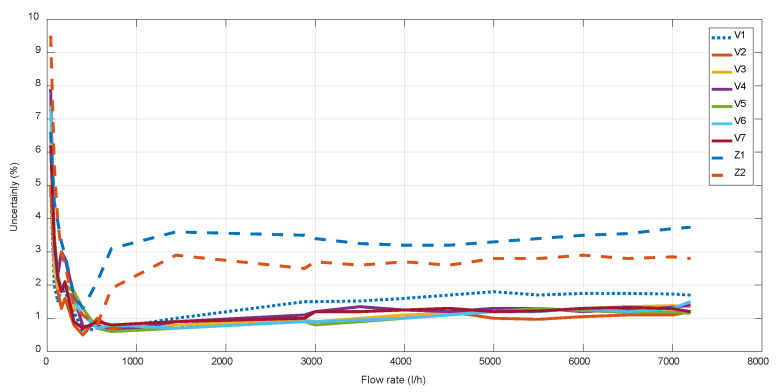
Uncertainty of different mechanical designs.

Figure 7 illustrates how Z-designs present a higher uncertainty, especially at high flow rates, because of a higher maximum *v* and/or a lower incidence angle.

**Figure 8:**
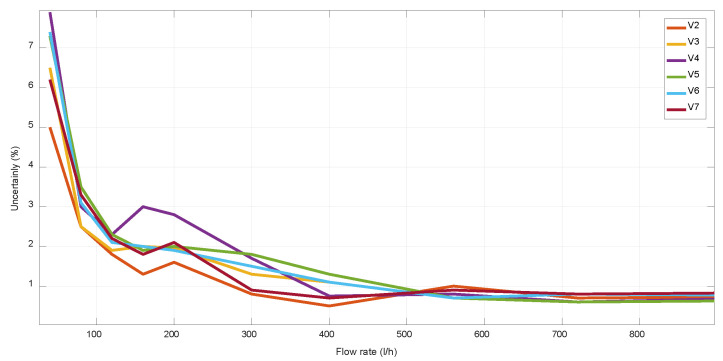
Uncertainty of different mechanical V-designs at low flow rates.

**Figure 9:**
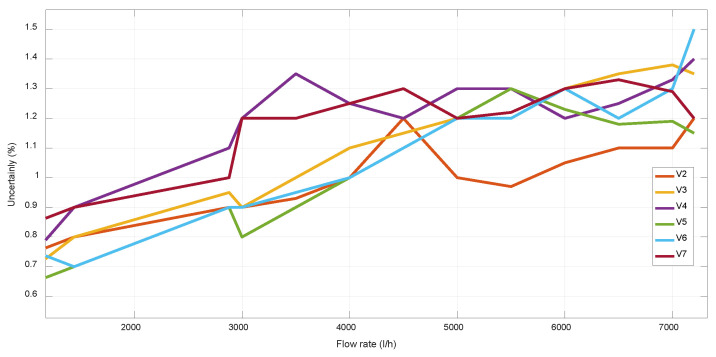
Uncertainty of different mechanical V-designs at high flow rates.

For V-designs, V1 presents the higher uncertainty at high flow rates, which is also due to its higher *v* range, as well as because its lower incidence angle provokes a higher beam deviation of Δ*x* ≈ 0.8mm. For the cases of V-designs with *α* = 63–67° (Figure 8 and Figure 9), a similar uncertainty was obtained for all of them, achieving the best results with V2, which has an intermediate value of the maximum *v* and the minimum DToF, as well as the optimal value of *α* = 65°.

**Figure 10:**
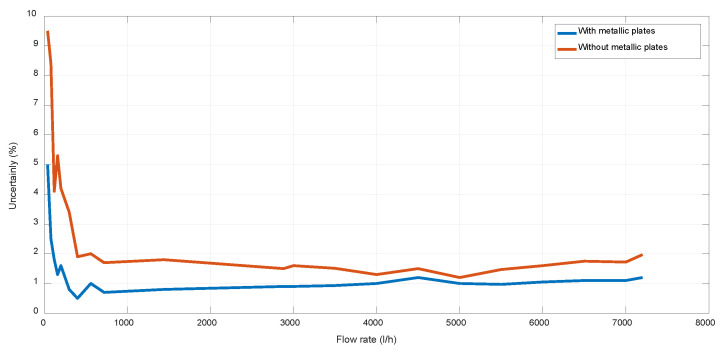
Uncertainty achieved with and without lamination.

Finally, with the objective of illustrating the benefits of using metallic separation plates for flow stabilization purposes, Figure 10 shows the uncertainty achieved with design V2 using three metallic plates (as all the designs illustrated in Figure 8 and Figure 9) and in the case of V2 without any lamination.

#### 2.2.3. Transducer Selection and Validation

In this stage, an experimental analysis was performed in order to select the nominal frequency of the ultrasonic transducers, which usually ranges from 200 kHz to 500 kHz for gas applications. First, by using a preliminary V-configuration pipe with an ultrasonic path length of 66 mm, transducers from Jiakang were selected over other manufacturers because of their performance regarding sensitivity. The main composition of these transducers is lead zirconium titanate, and they have been measured working at their radial vibration mode. Finally, the main features of these transducers (models PSC200K018102H3AD6-B1 at 200 kHz and PSC500K018099H2AD0-B1 at 500 kHz) are summarized in Table 2.

**Figure 11:**
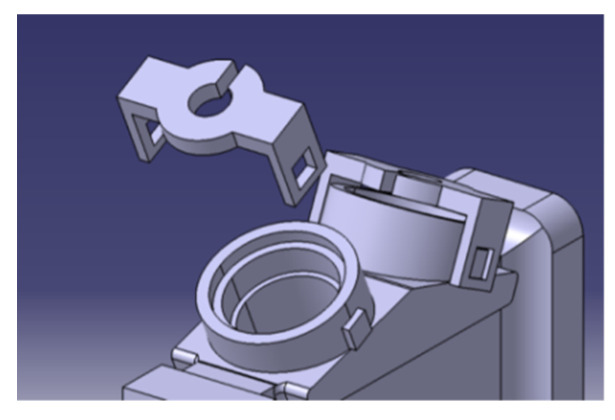
Attachment of the transducers on the pipe.

On the other hand, regarding the transducers’ attachment, they are installed in the holes designed for this purpose over the pipe and fixed with a piece that is attached to the sensor tube, as illustrated in Figure 11.

Regarding sensitivity, working at 200 kHz and with a driving input voltage of 3.3 V, the received amplitude signal was 9.6 mV (86.3 mV for a face-to-face case) and 3.4 mV at 500 kHz (39 mV for a face-to-face case). Thus, the attenuation in the air ranges between 42.7 dB/m at 200 kHz and 44.8 dB/m at 500 kHz, so working at lower frequencies results in a better option regarding signal attenuation. This increase in the dynamic range for the 200 kHz case will provide an additional benefit, especially in a gas medium, where the attenuation is increased in 14–23.5 dB, as described in Section 2.2.4.

Additionally, using the transducer at 200 kHz will provide other benefits, such as allowing for a lower sampling frequency, contributing to reducing the energy consumption, and providing a larger room to increase the gain of the pre-amplification stage, which is limited by its gain-bandwidth (G·BW) product, as will be described in Section 2.2.5.

The selected transducers have also been tested over the whole temperature range using a climatic chamber. First, 8 pairs at each frequency were experimentally tested in order to evaluate their zero-flow drift performance. In transit-time flow sensors, zero-flow drift is mainly due to the non-reciprocity of the piezoelectric transducers, i.e., they are non-identical because of the variations in different manufacturing parameters, such as disk thickness, disk permittivity, acoustic impedance, or coaxial cable length [33]. These non-reciprocity effects can be reduced by using different techniques at the electronics [32,34,35,36] or signal-processing levels [37].

**Figure 12:**
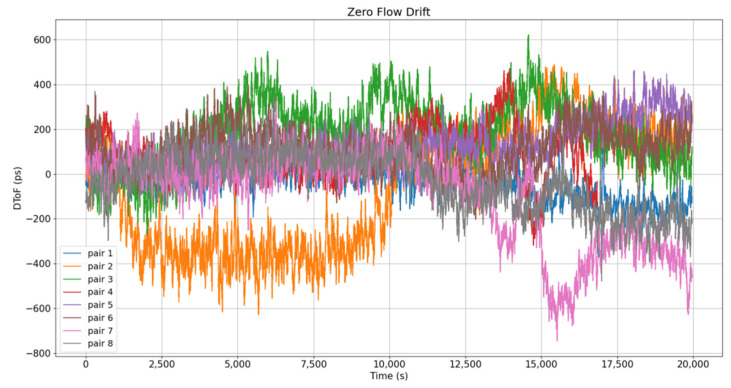
Zero-flow drift of 200 kHz transducers.

In this work, the zero-flow drift test was based on the measurement of DToF without the flow gradually along the temperature range (−10° to 40 °C), which is theoretically 0, in order to evaluate the transducers’ performance regarding temperature variations without the flow influence. Table 3 illustrates the maximum positive and negative values measured for each pair, leading to an absolute maximum zero-flow drift of <800 ps for 200 kHz and < 1200 ps for 500 kHz. These values also determine the minimum flow rate detectable by the sensor, corresponding to 1.3 L/h for 200 kHz and 2.1 L/h for 500 kHz. In addition, Table 3 shows the measured errors integrated over time. In general terms, it is possible to appreciate a better performance for the 200 kHz case, with an average of 37.25 ps of the integrated errors for the 8 pairs, lower than the average for the 500 kHz case, which results to 319.63 ps for the 8 tested pairs. Additionally, Figure 12 illustrates the obtained zero-flow drift for the 200 kHz case, where the axis x represents the test time as the temperature is gradually modified from −10° to 40 °C, i.e., 1 °C approximately every 400 s.

The transducers were also evaluated regarding the deviation between different pairs measuring the flow over the temperature. This analysis is crucial to the idea of avoiding an individual temperature calibration procedure for each sensor for different nominal flow rates and temperatures. Since this option is prohibitive for mass-production purposes, the idea would be to implement the calibration of each sensor only at the ambient temperature, so reducing the cost per unit.

**Figure 13:**
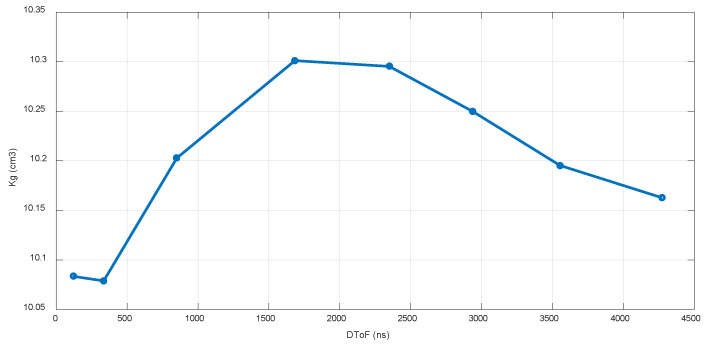
Piecewise linear calibration.

Thus, the calibration procedure is based on Equation (8), measuring Δ (DToF) for each flow rate specified by the standard in the range 40–7200 L/h and calculating a *kg* for a 0% error regarding the flow reference at 23 °C. In other words, *k_g_* is adjusted to obtain the same flow as the reference for each measured DToF. These nominal values for each pair *k_g_*-DToF are stored in the sensor memory, so any intermediate flow rate can be calibrated by interpolation with a piecewise linear conversion [38], as illustrated in Figure 13.

Regarding temperature compensation, it is inherently implemented using Equation (8), which is based on the measurement of the absolute ToFs, being inversely proportional to the temperature, from Equations (1), (2) and (6). Therefore, by exploiting the higher accuracy obtained using the selected HT-based correlation method, as described in Section 3, it would be possible to implement a cost-effective temperature compensation while avoiding the use of a temperature sensor, which implies a sensitive increase in the cost per sensor unit and power consumption.

**Figure 14:**
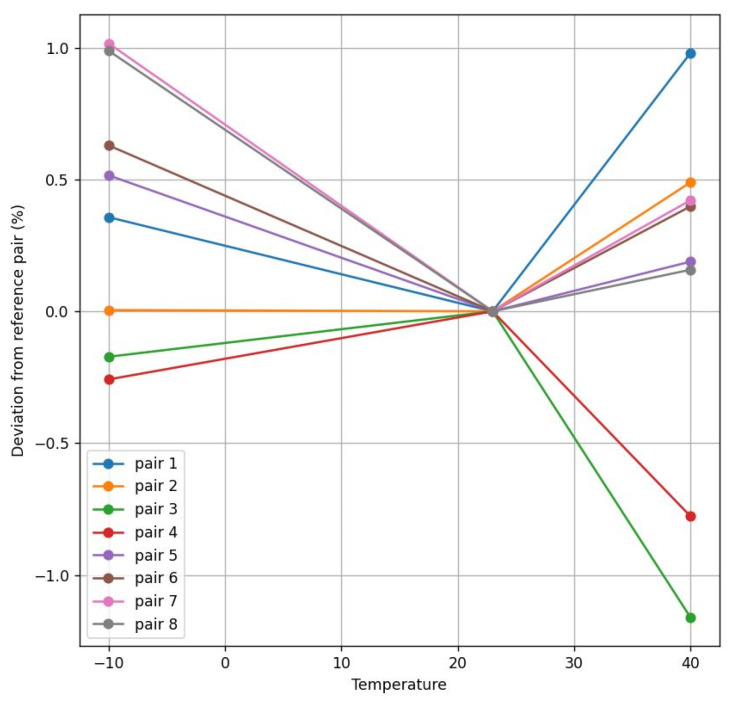
Flow deviation between 200 kHz pairs at 560 L/h.

**Figure 15:**
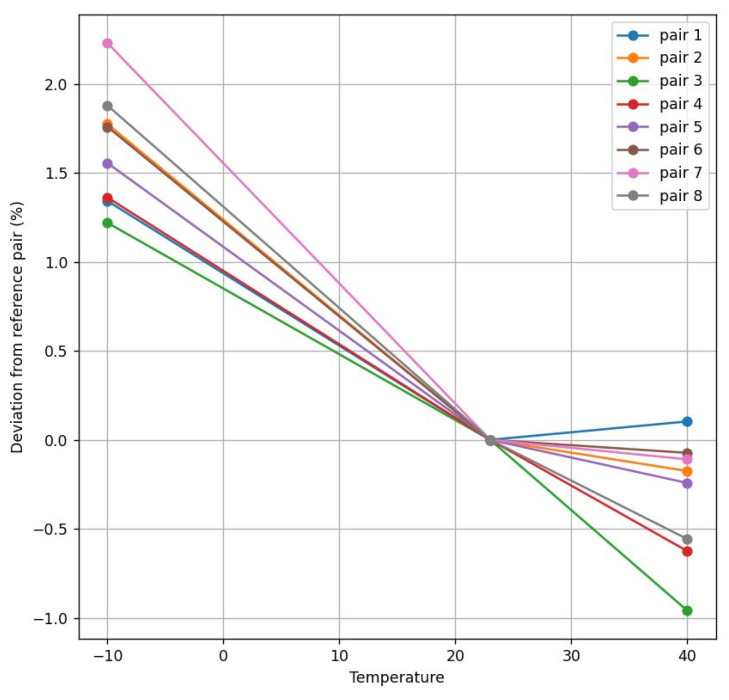
Flow deviation between 200 kHz pairs at 2000 L/h.

**Figure 16:**
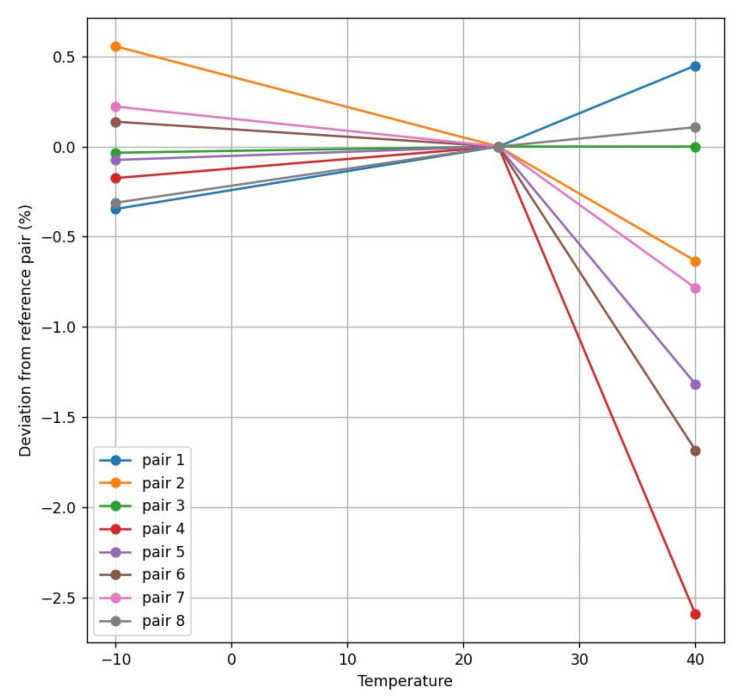
Flow deviation between 500 kHz pairs at 560 L/h.

**Figure 17:**
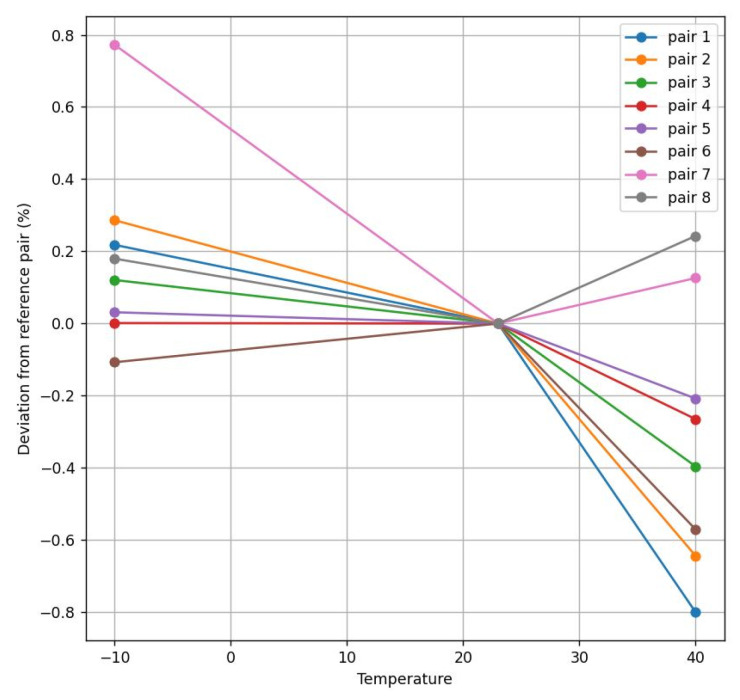
Flow deviation between 500 kHz pairs at 2000 L/h.

At both transducers’ frequencies, the obtained results are similar, as is illustrated in Figure 14, Figure 15, Figure 16 and Figure 17. These figures represent the deviation between different transducer pairs regarding the ambient temperature (i.e., considering the sensor is calibrated at 23 °C) for different flow rates (560 and 2000 L/h) and the extreme temperatures (−10° and 40 °C). Further, note that the results obtained for the 200 kHz case (with a maximum error of 1.07% for 560 L/h at 40 °C) provide a higher accuracy than those for the 500 kHz case (with a maximum of 1.52% for 560 L/h at 40 °C) in order to comply with the standard specifications, which require an error < 3% in the range of 40–600 L/h and <1.5% in the range of 600–7200 L/h.

Therefore, from the described experimental comparison with the 500 kHz case, a nominal frequency of 200 kHz was finally selected because of its properties regarding sensitivity (relaxing the requirements of the pre-amplification stage, thus reducing the hardware costs and the energy consumption), zero-flow drift performance, and flow measurement deviation between pairs.

#### 2.2.4. Sensor Configuration

This section describes the MSP430FR6043 (Texas Instruments, Dallas, TX, USA) parameters’ optimization, which was accomplished using the selected V2 design and the 200 kHz Jiakang transducer.

(a) *Transmit frequency and pattern option*

**Figure 18:**
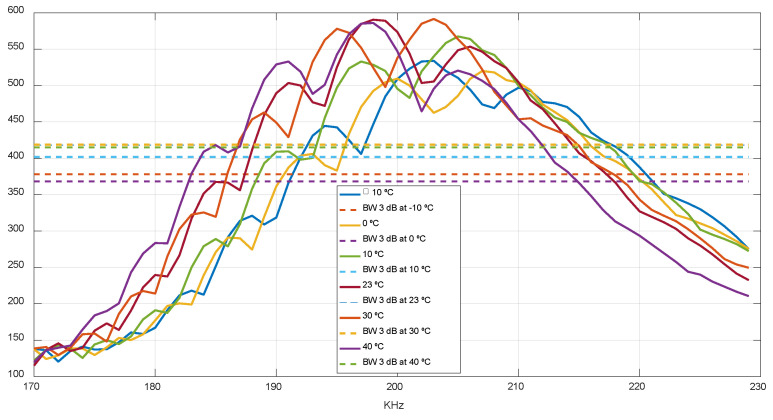
Frequency response at different temperatures.

Once the transducer frequency has been selected, it is possible to select between two different modes: single-tone and multi-tone. For the single-tone mode, pulses are generated at the nominal frequency, whereas for the multi-tone mode, pulses are generated starting at the *F*1 frequency and increasing to the *F*2 frequency, where *F*1 and *F*2 were selected as the 3 dB high-pass and low-pass frequencies of the transducer response. Figure 18 illustrates this frequency response at zero-flow conditions and different temperatures, where the Y axis represents the points of the ADC capture with a full scale of 2048 points, resulting in a 3 dB bandwidth of 197–212 kHz, which will be used to sweep the transmit frequency for the multi-tone mode.

**Figure 19:**
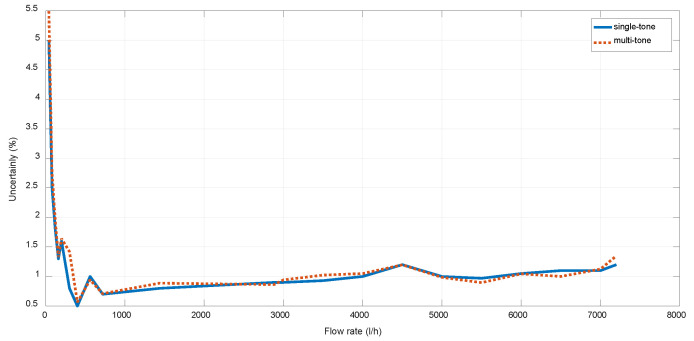
Uncertainty achieved for single-tone and multi-tone modes.

The single-tone mode provides a better performance if the frequency response is constant across temperature and flow conditions, implying a simpler solution, whereas the multi-tone mode provides a higher robustness against transducer frequency response variations along the entire temperature range [39] As illustrated in Figure 19, the obtained uncertainty is slightly lower for the single-tone mode at 23 °C and low flow rates, so it is the mode selected for the rest of test described in this paper. The multi-tone mode has been kept as a possible future alternative, in case the temperature-compensation method to be implemented does not provide the required accuracy.

(b) *Time between the pulse is transmitted, and the ADC starts to capture the signal*

In order to reduce power consumption, this time must be minimized, being limited by the minimum ToF, which is (from Equation (1)) 139.1 μs at the maximum flow rate (7200 L/h), with *c* = 435m/s and *v* = 10.27 m/s. From Equation (6), this minimum ToF will be reduced more at higher temperatures, resulting in μs 134.8 at 40 °C. Since, according to the manufacturer’s recommendation, the ADC needs a time of 50–60 μs before the signal is captured, this time was experimentally adjusted to 65 μs.

(c) *Number of pulses*

The number of transmitted pulses has to be enough to build the whole envelope of the received signal. For the V2 design, this minimum experimentally results in 8 pulses. The experimental variance obtained for a higher number of pulses is shown in Table 4 for a flow rate of 40 L/h, which is the most critical point to comply with the standard requirements. It is possible to observe how, by using more than 8 pulses, the accuracy is slightly improved. However, a value of 8 pulses was preliminarily selected in order to reduce the charge consumption, which is increased 0.0115 μA·s per each transmitted pulse.

Moreover, it is possible to add additional pulses to the transmitted pattern at the end of the frame with a phase shift of 180°. The function of this self-interference method (also called *stop pulses*) is to increase the ring down at the end of the ADC capture, by reducing the oscillations that the transducer maintains by inertia after receiving the pulses, which is a performance that will depend on the selected transducer [40]. This method to damp the ringing leads to a simpler implementation than those based on wideband transducers [41]. Thus, the use of these *stop pulses* improves the variance, as shown in Table 4, so a pattern based on 8 pulses and 2 *stop pulses* was finally selected, presenting the best results regarding the figure of merit (FoM) given by:

(14) $$FoM=\frac{1}{\sigma \cdot N\cdot Q_{1p}},$$

where *σ* is the obtained variance, *N* is the total number of pulses, and *Q*_1*p*_ is the charge consumption required per pulse (0.0115 μA·s). Similar conclusions were obtained for higher flows, so the minimum flow rate has been used as a reference, because is the most restricted case in terms of accuracy.

(d) *Upstream-to-downstream time*

The time between the transmissions of both transducers has been minimized in order to perform a complete flow measurement as fast as possible, with the objective of turning off the ultrasonic front-end after each measurement, in order to reduce the power consumption. For the V2 design, from Equations (2) and (6), the maximum ToF is 149.6 μs (at 7200 L/h and −10 °C), and the minimum ADC time capture recommended by the manufacturer is 250 μs (enough to convert a 10-pulse signal), so upstream-to-downstream time is set to 400 μs.

(e) *Gain*

**Figure 20:**
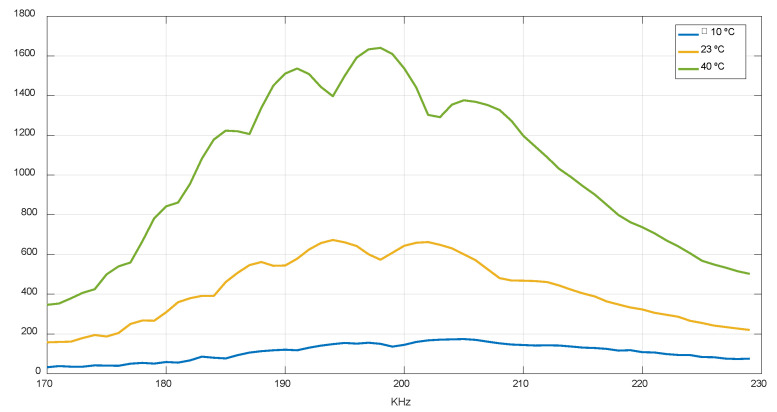
Amplitude received at different temperatures.

The PGA gain ranges between −6 dB and 30 dB. For the V2 design, in gas at an ambient temperature, this gain was set at 15 dB (14 dB higher than the air case), which has to be increased up to 24.5 dB in the worst case (−10 °C), so it is dynamically adjusted in the function of the received amplitude. Figure 20 shows the amplitude at ambient and extreme temperatures in a gas medium using a PGA gain of 15 dB.

(f) *ADC sampling frequency*

The ADC sampling frequency (*f_s_*) can be programmed in the range of 1–2 MHz. Using the minimum value of 1 MHz, it is possible to convert 5 samples per received pulse, which is enough to build the envelope signal and to estimate the ToF properly, without a difference regarding accuracy compared with sampling at 2 MHz. Thus, a *f_s_* of 1 MHz was selected, because higher sampling frequencies would lead to higher power consumption, as the ToF detection algorithm time would be increased as well.

#### 2.2.5. Hardware Design

**Figure 21:**
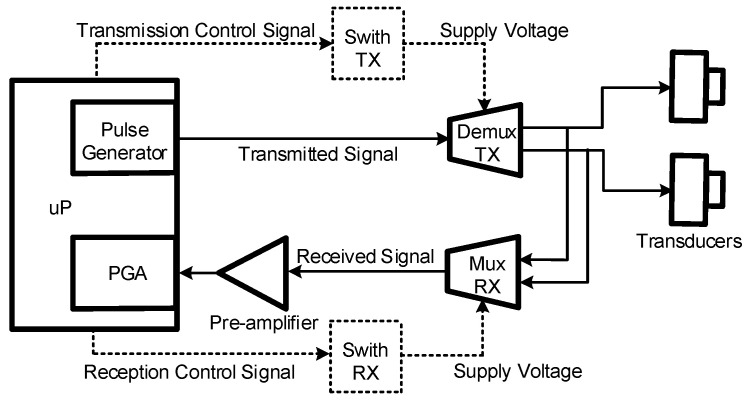
Hardware block diagram.

**Figure 22:**
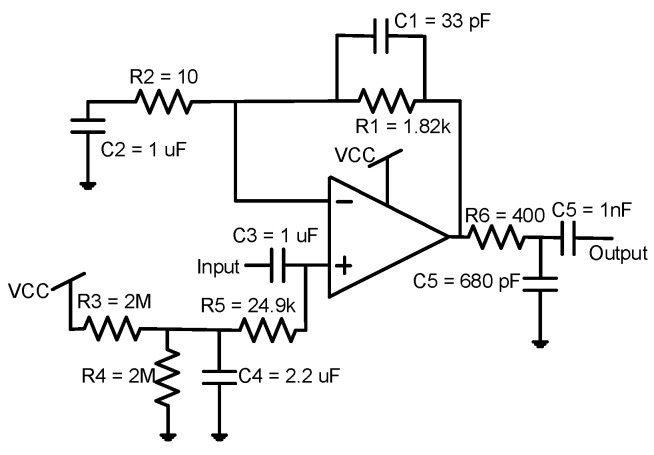
Pre-amplification schematic.

The electronic circuitry integrated into the sensor is based on COTS, specifically on the components illustrated in Figure 21 and described below:

- MSP430FR6043 (Texas Instruments, Dallas, TX, USA) ultrasonic front-end with an integrated microprocessor (uP), programmable pulse generator (PPG), and PGA.
- TPS22860 (Texas Instruments, Dallas, TX, USA) analog switches with time domain multiplexing to provide the supply voltage at the appropriate times.
- TS5A9411 (Texas Instruments, Dallas, TX, USA) multiplexers to connect the signals to their correct transmission or reception chains at the appropriate times.
- Pre-amplification stage based on the low-power operational amplifier OPA836 (Texas Instruments, Dallas, TX, USA) (Figure 22).

Therefore, the excitation signal (at 3.3V and 200 kHz) is generated by the PPG, with a low-impedance output driver (4 Ω), in order to not significantly affect to the impedance transducer, with a measured value of 449.3 Ω. In addition, the multiplexer manufacturer specifies a very low on-state resistance match between channels of 0.05 Ω, which should not affect reciprocity terms, according to the criteria regarding the matching of electronic components for reciprocal operation described in [36]. Finally, note that the employed multiplexer was also selected because of its ultra-low leakage current specification of 400 pA.

On the other hand, the pre-amplification circuit implements a gain of 45.2 dB, which is enough for accurate measurements for the case of higher attenuation (at −10 °C), where the PGA gain (with a maximum of 30 dB) has to be set at 24.5 dB, as previously described. Thus, the total gain is 45.2 dB + 24.5 dB = 69.7 dB in this case. In addition, several filtering options implemented in this stage were tested by modifying the capacitances *C*1 and *C*2. The objective of this analysis was to optimize the filter bandwidth in order to increase the available gain room, which is restricted by the G·BW product of 110 MHz specified by the pre-amplifier manufacturer and also by the minimum necessary gain at the pre-amplifier stage in the case of higher attenuation (69.7 dB − 30 dB = 39.7 dB). Note that for this application, bandwidth is not limited by the slew rate (SR) of the amplifier, because the maximum amplified voltage slope results in the order of 3–4 V/μs, much lower than the specified SR (560 V/μs). On the other hand, for the presented analysis, there is a trade-off between noise at the ADC input and distortion. Therefore, this study was focused on minimizing this noise without provoking distortion at the output.

**Figure 23:**
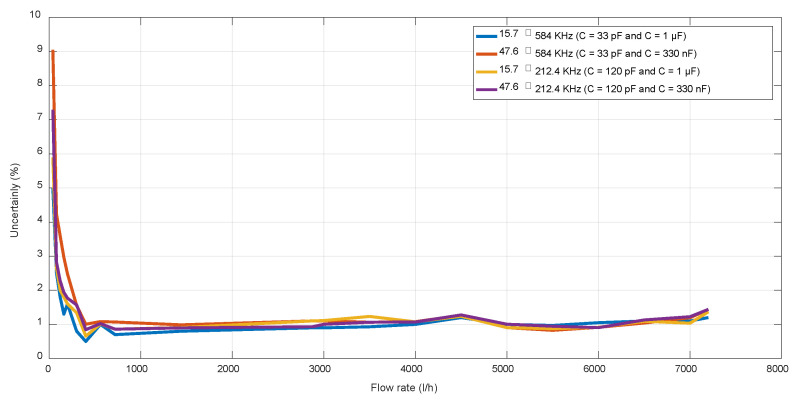
Uncertainty achieved with different filtering options.

The results derived from this analysis are illustrated in Figure 23. Four different filter bandwidth options were evaluated: 15.7–584 kHz (*C*1 = 33 pF and *C*2 = 1 μF), 15.7–212.4 kHz (*C*1 = 120 pF and *C*2 = 1 μF), 47.6–584 kHz (*C*1 = 33 pF and *C*2 = 330 nF), and 47.6–212.4 kHz (*C*1 = 120 pF and *C*2 = 330 nF). It is possible to observe how higher bandwidths benefit ToF detection, mainly at low flow rates. Therefore, a bandwidth of 568.3 kHz was selected, obtaining a G·BW = 103.4 MHz, close to the maximum of 110 MHz, so still with room to increase the pre-amplification gain, although this should not be necessary with a room of 30 dB − 24.5 dB = 5.5 dB with the PGA.

**Figure 24:**
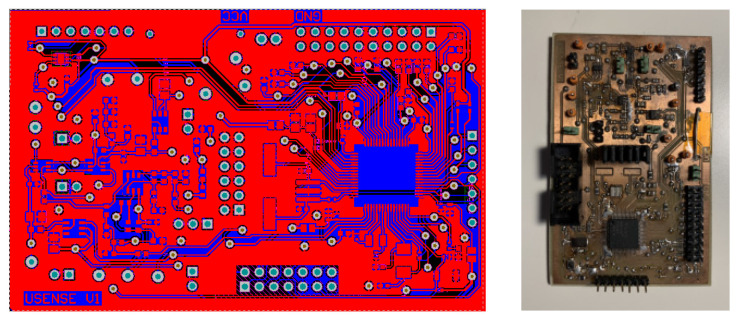
Designed and manufactured PCB.

Finally, the manufactured and experimentally characterized printed circuit board (PCB) is illustrated in Figure 24.

## 3. Results and Discussion

**Figure 25:**
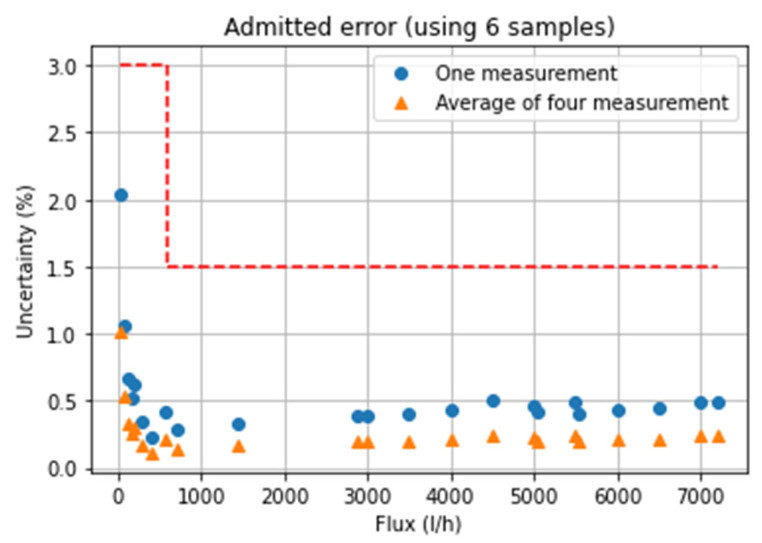
Achieved uncertainty.

The final design was tested after the integration of all the previously design parts, achieving the accuracy results presented in Figure 25. It is possible to appreciate how the standard EN14236 specifications are taken fully into account. These require an error (averaging six measurements) < 3% in the range of 40–600 L/h and <1.5% in the range of 600–7200 L/h. Specifically, the designed sensor presents a maximum error of 2.04% at 40 L/h. In Figure 25, the dash line corresponds to the admitted error, and the solid lines correspond to the achieved error after averaging six samples (as required by the standard), for the cases of one and four measurements of DToF per flow measurement.

**Figure 26:**
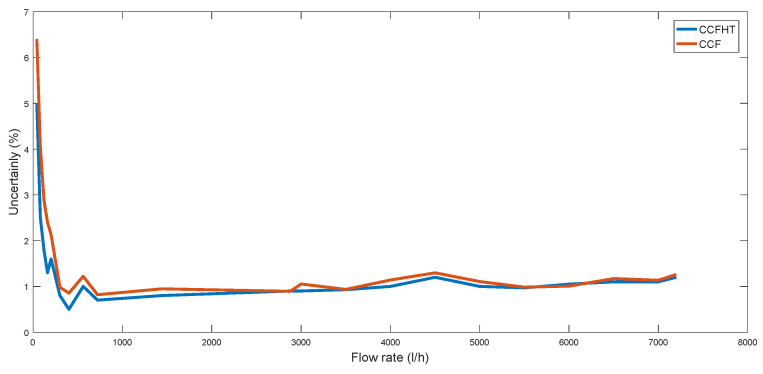
Uncertainty achieved for CCF and CCFHT cases.

Moreover, the measured power consumption resulted in 21.6 μA per flow measurement, lower than other commercial ultrasonic flow sensors, such as the Panasonic F9CM62A and the Maxim MAX35104, which report 25.7 μA and 31 μA, respectively. This minimized power consumption has been achieved from the sensor’s optimization, previously described in Section 2, which is realized at different levels, i.e., ultrasonic sensitivity optimization, sensor configuration, hardware design optimization, and also exploiting the accuracy provided by the correlation algorithm based on HT. Note that this total power consumption was measured for the proposed CCFHT-based method, with a data processing time of 16.1 ms. By performing a CCF-based algorithm, the measured total power consumption resulted in 19.6 μA, which provides a higher computing efficiency with a lower data-processing time per flow measurement (14.1 ms), because an additional processing time is necessary to previously implement the HT of the signals. However, regarding accuracy, by using a CCFHT-based implementation, the achieved uncertainty had a better result than the CCF case for the same mechanical design, as illustrated in Figure 26. It is possible to appreciate how the achieved uncertainty is up to 1.4% (at 40 L/h) enhanced for CCFHT case, improving the results especially at low flow rates.

Therefore, these results imply that it would be possible to perform a lower number of ToF detections per flow measurement in the CCFHT case in order to obtain the same accuracy results. For example, in the most critical case (at 40 L/h), an uncertainty of 5.0% is achieved for the CCFHT case and 6.4% for the CCF case. Assuming 5.02 = 6.40/√*x*, where *x* is the number of CCF-based ToF measurements, these accuracy results lead to the need to perform *x* = 1.6 measurements for each measurement using CCFHT, so it would lead to an effective power consumption of 1.6·19.6 μA = 31.4 μA, quite higher than the 21.6 μA achieved using the proposed CCFHT method. As such, it is possible to conclude that the proposed CCFHT-based method is more efficient taking both terms into account, i.e., accuracy and computational burden.

In terms of accuracy, the proposed sensor presents similar results compared with other previously published works, such as [2,3]. Although these ultrasonic gas flow sensors are focused on higher flow rate applications, they can be compared in the cases of their minimum flow rate, i.e., 10,000 L/h in [2] and 5000 L/h in [3]. Thereby, at ambient temperatures and using the six-measurement average required by the standard, [2] achieves an accuracy of 0.48% at 10,000 L/h, whereas the proposed sensor achieves 0.49% at 7200 L/h. Similarly, [3] presents an accuracy of 0.81% at 5000 L/h, and the proposed sensor achieves 0.41% at the same flow rate. Additionally, the proposed sensor is implemented using a cost-effective and high-energy efficiency solution compared with other works, which are implemented using power-hungry devices, as a FPGA in [2] or an external computer in [3].

Finally, a comparison in terms of accuracy with an ultrasonic gas flow sensor in a similar flow range [1] is presented in Table 5, showing the accuracy results for the flow rates given by [1]. In general, [1] achieves a better accuracy at low flow rates and a worse accuracy at high flow rates, although both sensors fully comply with the standard requirements. However, note that [1] does not provide results in terms of flow measurement drift for different temperatures or in terms of energy consumption. Additionally, regarding zero-flow drift, [1] presents an error of ±5 ns, which is higher than the ±0.7 ns achieved in this work and reported in Section 2.2.3.

## 4. Conclusions

A fully designed ultrasonic cross-correlation based gas flow sensor has been presented in this paper. All the sensor design stages (at the mechanical, electronic, and signal-processing levels) have been developed and experimentally validated, obtaining an accuracy performance that completely fulfills the standard requirements. The proposed sensor has been optimized in terms of accuracy and power consumption by the analysis, validation, and integration of each design part. This optimization has been performed with regard to the selection of the mechanical dimensions and materials of the pipe, the piezoelectric transducers and their configuration, the electronics hardware, and the ToF detection algorithm based on the implementation of the cross-correlation of the received signals using the Hilbert Transform. As a result, the proposed sensor is placed into the state of the art of ultrasonic gas flow sensors in terms of accuracy, and it provides additional features when compared with most of the previously published works, such as lower power consumption and hardware complexity minimization. Additionally, by exploiting this enhanced accuracy, the measurement of the absolute ToFs can be used to compensate for the errors caused by temperature variations in a future implementation. The presented enhancement in terms of accuracy and energy consumption, with its low-cost and low-maintenance features, lead the proposed sensor to be considered as a competitive solution to be integrated into smart IoT sensor networks.

## Figures and Tables

**Table 1:** Manufactured and tested mechanical designs.

Design	Length (mm)	Width (mm)	High (mm)	Section (mm^2^)	Angle (°)	Minimum DToF (ns)	Maximum *v* (m/s)
V1	73.8	9.9	19.2	190.55	45	51.71	10.50
V2	61.1	8.3	23.6	194.75	65	25.04	10.27
V3	58.8	8.5	22.3	189.49	67	22.90	10.55
V4	54.7	10.2	19.1	195.30	63	24.00	10.24
V5	63.4	8.1	23.7	191.13	65	26.48	10.46
V6	58.6	9.1	22.5	203.39	65	23.00	9.83
V7	56.6	8.1	20.7	166.32	65	27.17	12.02
Z1	54.8	5.0	36.0	180.21	60	28.72	11.10
Z2	50.1	6.3	30.6	192.98	58	25.96	10.36

**Table 2:** Features of the selected transducers.

	200 kHz	500 kHz
Diameter (mm)	14.1	12
Static capacitance (pF)	2000 ± 20%	500 ± 10%
Signal sensitivity (mV)	15	50
Effective bandwidth (kHz)	±10	-
Tolerance pressure (MPa)	0.5	-
Directive property (°)	14 ± 2	6
Operating temperature range (°C)	−35/+70	−35/+70

**Table 3:** Zero-flow drift results.

Pair	Positive Drift at 200 kHz (ps)	Negative Drift at 200 kHz (ps)	Positive Drift at 500 kHz (ps)	Negative Drift at 500 kHz (ps)	Integrated Error at 200 kHz (ps)	Integrated Error at 500 kHz (ps)
1	339	−376	779	−644	−39	118
2	493	−635	538	−428	−60	−141
3	627	−271	792	−275	192	180
4	479	−326	1086	−322	92	649
5	466	−132	1191	−136	128	921
6	409	−200	651	−208	113	86
7	338	−752	1016	−759	−97	417
8	351	−483	849	−487	−31	327

**Table 4:** Comparison of pattern options for a flow rate of 40 L/h.

Number of Pulses	Number of Stop Pulses	Variance	FoM
8	2	3.78 × 10^−19^	2.30 × 10^19^
8	1	6.46 × 10^−19^	1.49 × 10^19^
8	0	5.80 × 10^−19^	1.87 × 10^19^
10	0	5.11 × 10^−19^	1.70 × 10^19^
12	0	3.52 × 10^−19^	2.06 × 10^19^
14	0	3.40 × 10^−19^	1.83 × 10^19^

**Table 5:** Accuracy results comparison.

	40 L/h	1440–1600 L/h	4000 L/h
Proposed work	2.04%	0.33	0.41%
[1]	1.67%	1.09%	0.74%

## Data Availability

Not applicable.
